# Gut colonization by a novel *Clostridium* species is associated with the onset of epizootic rabbit enteropathy

**DOI:** 10.1186/s13567-018-0617-8

**Published:** 2018-12-20

**Authors:** Ana Djukovic, Marc Garcia-Garcera, Eugenio Martínez-Paredes, Sandrine Isaac, Alejandro Artacho, Jorge Martínez, Carles Ubeda

**Affiliations:** 1 Departamento de Genómica y Salud, Centro Superior de Investigación en Salud Pública – FISABIO, Avenida de Cataluña, 21, 46020 Valencia, Valencia Spain; 2 Department of Fundamental Microbiology, University of Lausanne, 1006 Lausanne, Switzerland; 3 Instituto de Ciencia y Tecnología Animal, Universitat Politècnica de Valencia, Camino de Vera, s/n., 46022 Valencia, Valencia Spain; 4 Departament de Sanitat i Anatomia Animals, Universitat Autònoma de Barcelona, Bellaterra, 08193 Cerdanyola del Vallés, Spain; 5 CIBER en Epidemiología y Salud Pública, 28029 Madrid, Spain

## Abstract

Epizootic rabbit enteropathy (ERE) represents one of the most devastating diseases affecting rabbit farms. Previous studies showing transmissibility of disease symptoms through oral inoculation of intestinal contents from sick animals suggested a bacterial infectious origin for ERE. However, no etiological agent has been identified yet. On the other hand, ERE is associated with major changes in intestinal microbial communities, pinpointing dysbiosis as an alternative cause for the disease. To better understand the role of intestinal bacteria in ERE development, we have performed a prospective longitudinal study in which intestinal samples collected from the same animals before, during and after disease onset were analyzed using high-throughput sequencing. Changes in hundreds of bacterial groups were detected after the initiation of ERE. In contrast, before ERE onset, the microbiota from rabbits that developed ERE did not differ from those that remained healthy. Notably, an expansion of a single novel *Clostridium* species (*Clostridium cuniculi*) was detected the day of ERE onset. *C. cuniculi* encodes several putative toxins and it is phylogenetically related to the two well-characterized pathogens *C. botulinum* and *C. perfringens*. Our results are consistent with a bacterial infectious origin of ERE and discard dysbiosis as the initial trigger of the disease. Although experimental validation is required, results derived from sequencing analysis, propose a key role of *C. cuniculi* in ERE initiation.

**Electronic supplementary material:**

The online version of this article (10.1186/s13567-018-0617-8) contains supplementary material, which is available to authorized users.

## Introduction

Epizootic rabbit enteropathy (ERE) is an intestinal disorder that naturally and frequently occurs in farm rabbits (between 11 and 65% of rabbits in a farm can develop the disease [1, 2]). In addition, ERE is associated with high mortality rates (30–95% of ERE rabbits) [3], which makes ERE one of the most devastating diseases in rabbit farms. ERE frequently starts within the first 2 weeks after weaning, a period that is associated with changes in intestinal commensal microbial communities and microbiota instability [4]. The onset of the disease can be easily identified by a sharp decrease in food consumption (first sign of ERE) [5, 6]. Other signs and gross lesions, including prostration, diarrhea, caecal impaction, stomach dilation, and increased colonic mucus secretion, follow this. Although the etiology of ERE is unknown, several studies have suggested an important role of intestinal bacterial populations in the initiation of ERE [1, 7, 8]. Licois et al. were able to reproduce the symptoms of the disease by orally inoculating intestinal contents from ERE rabbits to specific-pathogen-free (SPF) rabbits [8]. Additional studies using sucrose gradient fractions of intestinal contents as the inoculating material discarded a role of virus or parasites in the initiation of ERE [7, 9], suggesting a bacterial origin of ERE. Subsequent studies have been performed in order to identify an etiological agent of the disease. Marlier et al. repeatedly isolated *Clostridium perfringens* and non-pathogenic *Escherichia coli* at high faecal counts from animals that developed ERE [1]. Moreover, a high correlation between the presence of *C. perfringens* alpha toxin and ERE gross lesions was identified. However, administration of the isolated bacterial strains did not reproduce ERE symptoms [1]. Following studies applied 16S rRNA high-throughput sequencing in order to characterize differences in intestinal bacterial populations between ERE and healthy rabbits [10, 11]. Huybens et al. analyzed the microbiota of several reference inocula and virulence fractions known to reproduce ERE and compared it with the microbiota from SPF rabbits [10]. However, their analysis failed to identify a single bacterial genus that would discriminate ERE samples from healthy samples. On the other hand, Baüerl et al. identified changes in multiple bacterial taxa in the caecal microbiota of rabbits that had developed ERE. These changes included the expansion of the *Clostridium* genus and γ-Proteobacteria and the reduction of specific commensals such as *Alistipes* and *Ruminococcus*. Thus, besides a bacterial infectious origin, dysbiosis could be playing a major role in the development of ERE. Nevertheless, in both studies, the microbiota composition of rabbits was evaluated after the onset of the disease. However, pathological alterations occurring during the disease can alter the microbiota [12]. Thus, most changes detected after onset could be merely consequence of the disease, which difficults the identification of intestinal bacterial populations that could be the key in the disease etiology. To circumvent this problem, we have performed a longitudinal study of caecotroph samples in order to evaluate, within the same rabbit, microbiota changes occurring before, during and after the onset of the disease. Notably, changes in hundreds of different bacterial groups could be detected after ERE onset. In contrast, exclusively one bacterial change (i.e. expansion of a novel species from the genus *Clostridium*) could be detected at the very beginning of the disease onset, suggesting a key role of this particular species in ERE development.

## Materials and methods

### Rabbit model and housing conditions

The study was performed on 33 rabbits from a line raised at the Polytechnic University of Valencia (Spain) resulting from a cross between New Zealand White and California rabbits. Animals were weaned at 30 days of age and housed in separate individual cages where they had ad libitum access to food and water. The food that was used in the diet was standard, antibiotic-free and coccidiostatic-free (Cunicebal Retirada, Nanta, Spain). Three independent experiments were performed. The number of animals per experiment varies due to the different number of cages or litters available. All experiments consisted on monitoring ERE signs and measuring the food consumption. During the first experiment 2 out of 9 animals developed ERE after weaning. In the second experiment 5 out of 14 rabbits developed ERE, and in the last experiment, 4 out of 10 rabbits developed ERE. Caecotroph samples from all experiments were analyzed together in the longitudinal analysis presented in the results. For each experiment, rabbits from different maternal origins were used (*N* = 2–4 litters per experiment).

### Identification of rabbits developing ERE and defining the day of ERE onset

A sharp decrease in food consumption is considered the first sign that can be detected in rabbits that develop ERE [5, 6]. For this reason, food consumption was measured daily in order to detect the onset of the disease. The day of disease onset was considered to be that one in which a rabbit diminished food consumption > 50% compared to the previous day. The threshold of 50% was chosen since a few healthy rabbits diminished food consumption on specific days (up to 46%, Additional file 1) without developing any ERE signs. On the contrary, ERE rabbits developed ERE compatible signs (prostration, diarrhea, tympanism) following the detected sharp decrease in food intake (Additional file 2). Moreover, none of the ERE rabbits increased food intake after ERE initiation (Additional file 3), while those healthy rabbits that diminished food consumption on a particular day for an undetermined reason, later recovered their food intake (Additional file 3). For ethical reasons, rabbits developing ERE were sacrificed when they ate during two consecutive days less than 10 g of food, except for four rabbits, which died of ERE symptoms before fulfilling this criterion. Healthy rabbits were followed for an additional week before euthanasia (total of 3 weeks after weaning) to ensure that they would not develop any ERE compatible signs.

To confirm ERE development, a necropsy was performed on all rabbits. This necropsy was performed by co-author Jorge Martínez, a veterinarian specialist in anatomical pathology (Diplomate by the European College of Veterinary Pathologists). Examination was performed in order to identify gross lesions compatible with ERE: distension of the stomach and intestine, caecum filled with gas, liquid or impacted with solid/dry content, and presence of abundant quantities of mucus in the colon (Additional file 2). Gross lesions compatible with ERE were observed in all rabbits that developed a sharp decrease in food consumption and ERE compatible signs (prostration, diarrhea) (Additional file 2). However, no lesions suggesting the presence of other intestinal disorders or other systemic diseases were detected in any of the ERE rabbits.

### Sample collection and DNA extraction

In order to assess microbiota composition of rabbits, caecotrophs were collected the day of weaning, the day after weaning and then every other day during the next 20 days. Caecotrophs are soft feces excreted by rabbits that have a similar chemical and microbiological composition to that of the caecal contents, where major pathological changes occur during ERE [13]. Caecotroph samples were collected using polyvinyl chloride (PVC) neck collars to prevent their ingestion by rabbits. Caecotrophs were kept at 4 °C during approximately 3 h and aliquots of these caecotroph samples (approximately 200 mg) were stored at −80 °C afterwards. Due to the symptomatology or rabbit death, we could only obtain caecotroph samples after ERE onset from 7 out of the 11 ERE rabbits. For comparison, we did also analyze caecotroph samples from 10 healthy littermate controls. For comparison with a mature adult microbiota (Figure 2), caecotroph samples from 4 mothers were collected the day of weaning and 7 days after weaning. DNA was extracted using a QIAamp^®^ DNA Stool Mini kit (QIAGEN) with a previous step of mechanical disruption to improve cell lysis as we have previously described [14]. Briefly, cells were resuspended in 1.4 mL of ASL buffer and 500 μL of 0.1 mm glass beads and tubes were vortex at maximum speed for 5 min prior to the initial incubation for heat and chemical lysis at 95 °C for 7 min. Subsequent steps of the DNA extraction followed the QIAamp kit protocol.

### 16S rRNA gene amplification and high-throughput sequencing

The V3–V4 region of the 16S rRNA gene was amplified and sequenced using the MiSeq platform from Illumina, as described in the manual for “16S Metagenomic Sequencing Library Preparation” of the MiSeq platform (Illumina). Briefly, for each sample, a 25 μL reaction was prepared containing 12.5 ng of DNA, 12.5 μL 2× KAPA HiFi Hot Start Mix, and 0.2 mM of primers. Water was added to complete the volume of the reaction. Cycling conditions were 95 °C for 3 min, and 25 cycles of 95 °C for 30 s, 55 °C for 30 s and 72 °C for 30 s, and a final elongation cycle at 72 °C for 5 min. The amplification was confirmed through electrophoresis by loading 4 μL of the PCR reaction on a 1.6% agarose gel. Subsequently, the PCR product was purified with the AMPure XP beads as described in the Illumina protocol. Next, a limited-cycle PCR reaction was performed to amplify the DNA and add index sequences on both ends of the DNA, thus enabling dual-indexed sequencing of pooled libraries. Index PCR consisted of a 50 μL reaction containing 5 μL of the DNA obtained from the previous PCR, 25 μL of 2× KAPA HiFi Hot Start Mix, and 5 μL of forward and reverse indexed primers. Temperature conditions were the same as for the first reaction, but the number of cycles was reduced to 8. The obtained PCR product was purified with the AMPure XP beads following the manufacture’s protocol. An equal amount of the purified DNA was taken from each sample for pooling. Each pool of samples (*N* = 96) was sequenced following Illumina recommendations.

### 16S rRNA sequencing analysis

Sequences were processed using Mothur v1.35 [15] as we have previously described [14], with some modifications. Initial trimming by quality was performed on paired ends of sequences before joining them into a single read. Parameters used for trimming included elimination of sequences shorter than 200 bp or that contained homopolymers longer than 8 bp or undetermined bases. Using the base quality scores, which range from 0 to 40 (0 being ambiguous base), sequences were trimmed using a sliding-window technique, such that the minimum mean quality score over a window of 50 bases never dropped below 25. Sequences were trimmed from the 3′ end until this criterion was met. Sequences were aligned to the 16S rRNA gene using as a template the SILVA reference alignment. Potential chimeric sequences were removed using Uchime algorithm. To minimize the effect of pyrosequencing errors in overestimating microbial diversity [16], rare abundance sequences that differ in up to two nucleotides from a high abundant sequence were merged to the high abundant sequence using the pre.cluster option in Mothur. An average of 17,297 sequences were obtained per sample. Since different number of sequences per sample could lead to a different diversity (i.e. more Operational Taxonomic Units—OTUs could be obtained in those samples with higher coverage), in order to compare the diversity of different caecotroph samples, we rarefied all samples to the number of sequences obtained in the sample with the lowest number of sequences (10 997). In other words, 10 997 sequences were randomly selected from each sample for subsequent analysis: taxonomic characterization and OTUs identification. Sequences with distance-based similarity of 97% or higher were grouped into the same OTU using the VSEARCH abundance based greedy clustering (AGC) method. OTU-based microbial diversity was estimated by calculating the Shannon diversity index. Each sequence was classified using the Bayesian classifier algorithm with the bootstrap cutoff of 60% [17]. In most cases classification could be assigned to the genus level. When it was not possible to classify a sequence to a certain taxonomic level, it was assigned as “Unclassified” followed by the upper taxonomic level. Sequences have been deposited in NCBI under the accession number PRJNA509120.

### Principal coordinate of analysis

In order to compare the overall microbiota similarity between different caecotroph samples, we calculated for every pair of samples the UniFrac phylogenetic distance. This distance was calculated by generating a phylogenetic tree containing all the 16S rRNA representative sequences of all the OTUs identified in the samples under comparison. Subsequently, the UniFrac distance was calculated as the fraction of the total branch length of the tree, which is not shared between the two samples under comparison. UniFrac distance values range from 0 to 1, being 1 the distance obtained between a pair of samples that do not share any branch of the phylogenetic tree (their microbiota is totally different and do not share any bacterial lineage), and 0 the distance obtained between a pair of samples that have exactly the same microbiota. To perform UniFrac analysis we first inferred a phylogenetic tree using clearcut, on the 16S rRNA sequence alignment generated by Mothur. Both unweighted UniFrac, which tabulates the presence or absence, but not the proportion, of different bacterial lineages within the tree, and weighted UniFrac, which takes into account the proportion of each bacterial lineage in each sample, were run using Mothur. Principal Coordinates of Analysis were performed on the resulting distance matrices with Mothur.

### Isolation of *Clostridium cuniculi*

**Figure 1:**
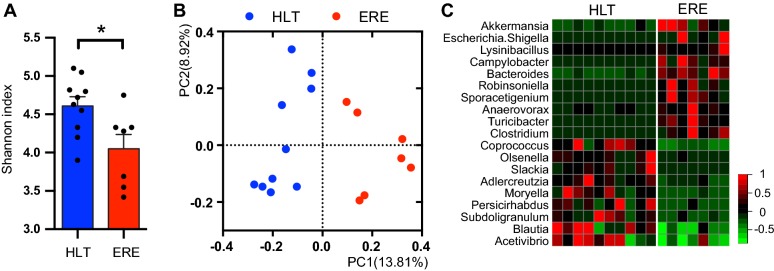
**ERE is characterized by dysbiosis of the intestinal microbiota after disease onset.** Microbiota composition was analyzed in caecotrophs samples collected from rabbits with ERE (1–3 days after ERE initiation) and compared with those collected from healthy (HLT) age-matched littermate controls. **A** The Shannon index of the identified OTUs for each sample and mean ± SEM for each group was calculated and plotted (*p < 0.05, Wilcoxon two-sided test). **B** PCoA from the unweighted UniFrac values calculated between all the samples. The microbiota of samples collected after ERE onset was significantly different from samples of healthy controls (AMOVA, *p* < 0.001). **C** Heatmap representing the standard deviation from the mean of the relative abundance of statistically significantly different genera between rabbits with ERE and HLT littermate controls (Wilcoxon two-sided test, *p* < 0.05, FDR < 0.1). Red colors represent values above the mean and green colors represent values below the mean. *N* = 10 for the HLT group and 7 for the ERE group (caecotrophs samples could not be collected from 4 ERE rabbits due to the symptomatology or rabbit death).

The *C. cuniculi* strain was isolated from a −80 °C frozen aliquot of a caecotroph sample collected (as described in the section “Sample collection and DNA extraction”) from one of the rabbits suffering from ERE indicated in Figure 1. The chosen sample was collected after ERE onset, and was characterized by 16s rRNA high-throughput sequencing as the one that contains the highest abundance of the OTU172 (the *Clostridium* OTU of interest, later defined as *C. cuniculi*). An aliquot of the chosen caecotroph sample was thawed under anaerobic conditions. 10-fold dilutions of the sample in pre-reduced phosphate buffer saline (PBS) were plated on TSA blood agar plates and incubated for 48 h under anaerobic conditions at 37 °C. Subsequently, a colony PCR using specific primers for the OTU172 was performed, as described below.

Specific primers were designed using the Primrose program. Briefly, a FASTA file with 16S rDNA sequences that are representative for every OTU detected in the caecotroph sample used for isolation were utilized to construct a database, which is used for the design of primers. This approach allows to select primers specific for the 16S rRNA sequence of the desired OTU, and that will not amplify any other OTUs present in the sample used for isolation. The maximum length of the primers was set to 20 nucleotides, while no ambiguous nucleotides were accepted. This analysis led to the design of the pair of primers: OTU172-F: AGGCGGACTTTTAAGTGAGAT and OTU172-R: CGTCAGTTACAGTCCAGAGAAT.

For each colony PCR, a 25 µL reaction was prepared containing: 1 μL of 1 bacterial colony resuspended in 10 μL of PBS, 2.5 µL 10× Standard Taq Reaction Buffer (New England BioLabs), 0.25 mM of deoxynucleotide triphosphates (dNTPs), 2.5 U of Taq DNA Polymerase (New England BioLabs) and 0.2 mM of primers. Cycling conditions were 94 °C for 5 min, and 35 cycles of 94 °C for 30 s, 56 °C for 30 s and 68 °C for 30 s, and a final elongation cycle at 68 °C for 5 min. Positive colonies were re-amplified using the primers used for 16S high-throughput sequencing, as described above. Sequences of amplified products were obtained through Sanger reaction and capillary electrophoresis. The obtained sequences were compared with the sequences that define the OTU172 using blastn in order to verify that the isolated colony was indeed the OTU172 (100% similarity to sequences contained within the OTU172). The colony that was identified as the OTU172 was regrown on TSA blood agar plates for 48 h under anaerobic conditions. Subsequently, the bacterial culture was resuspended in PBS containing 15% glycerol and stored at −80 °C.

### Experimental reproduction of ERE

Intestinal contents (caecum and small intestine) from 4 rabbits with ERE were diluted 1:2 in phosphate buffer saline containing 15% glycerol and 0.1% cysteine (previously reduced overnight in an anaerobic chamber) to preserve bacterial viability. Resuspended intestinal contents were frozen at −80 °C and thawed the day of inoculation. *C. cuniculi* was grown on TSA blood agar plates for 48 h and resuspended, the day of the inoculation, in pre-reduced PBS-glycerol-cysteine at a concentration of 10^7^ CFUs/mL. 5–6 weeks old SPF rabbits (*N* = 10 per group) were nasogastrically inoculated with either 1 mL of the resuspended intestinal contents, *C. cuniculi* or PBS-glycerol-cysteine (uninfected control). 2 rabbits were housed per cage. Rabbits from different groups were located in different rooms in order to avoid contamination.

### Sequencing of the *C. cuniculi* genome

To determine genes and functions encoded by *C. cuniculi*, genomic sequencing was performed. The isolated strain was grown on TSA blood agar in anaerobic conditions at 37 °C. Colonies were collected and resuspended in 1 mL of PBS. Bacterial cells were centrifuged at 13 000* g* during 20 min and supernatant was discarded. DNA was extracted with the QIAamp ^®^ Fast DNA Stool Mini kit as described previously.

Genomic DNA was prepared for sequencing using Nextera XT DNA Sample Preparation Kit. Briefly, 5 μL of Amplicon Tagment Mix was added to 10 μL of Tagment DNA Buffer. The amount of DNA added to the mix was 1 ng. The mix was incubated at 55 °C during 5 min. Neutralization of the reaction was achieved by adding 5 μL of Neutralize Tagment Buffer. For the step of amplification, 15 μL of Nextera PCR Mix was added. The cycling conditions were 72 °C during 3 min, 95 °C 30 s followed by 12 cycles of 95 °C during 10 s, 55 °C 30 s and 72 °C 30 s. A final elongation step was performed at 72 °C during 5 min. The obtained PCR product was purified with the AMPure XP Beads as described in the manufacturer’s protocol. Sequencing was performed using the MiSeq platform following Illumina recommendations. The genome sequence has been deposited in NCBI under the accession number PRJNA509119.

### Analysis of the *C. cuniculi* genome

Characterization of the strain of interest included the assembly and annotation of the data obtained by genome sequencing, evolutionary placement of the strain into the Clostridiales order, identification of the species to which it belongs, and functional annotation of the identified genes. First, sequences obtained by Illumina were processed in order to eliminate bad quality sequences. Briefly, adapter sequences were removed from the raw data using Cutadapt v. 1.12 [18]. Quality filtering was performed using UrQt v. 1.0.18 [19]. Only reads with a size of 75 bp or higher were further processed to avoid possible misclassification of short reads. Cleaned genomic data was assembled with SPAdes v. 3.7.1 [20] using the “careful” algorithm to improve the contig reconstruction. SPAdes resulted in a draft genome assembly of 160 contigs, of which 106 had a contig length larger than 1000bps. The average base pair coverage was 27.17 ± 2.97 X. Open-reading frames (ORFs) were identified and annotated using PROKKA v. 1.12 [21]. To maximize the annotation, ORFs were translated into amino acids and queried against 3 independent databases: Pfam v.27.0 [22], EggNOG v.4.5 [23], and KEGG [24]. Annotation was performed using HMMer v.3.1.2 (E value < 0.05, minimum coverage 0.5) [25].

Based on a recently published article [26] the genome draft of the isolate was compared against the reference genomes of the Clostridiales order, obtained from the GenBank RefSeq complete genome database (last accessed July 2018). To do so, we first constructed the core-genome of 98 genomes classified as Clostridiales. To build it, we started by assigning relations of orthology between pairs of genomes. Orthologs were defined as the bidirectional best hits between pairs of genes, using end-gap free global alignment (as in Touchon et al. [27]). Hits with less than 40% similarity in amino acid sequence or more than 20% difference in protein length were discarded. A total of 114 core-genes were identified for the whole Clostridiales order. A sequence concatenate of the core genome at amino acid level was built and aligned using Mafft v.7.15 [28]. Predicted genes from the isolate were aligned using Mafft against the Multiple Sequence Alignment concatenate. The Clostridiales phylogeny was reconstructed with IQ-tree using the concatenate multiple protein sequence alignment of the core-genes [29]. An outgroup of unrelated species was included to root the tree. Evolutionary placement algorithm was performed to locate the most probable phylogenetic location of the isolated strain. The best phylogenetic location was considered only when the Maximum Likelihood Weight was higher than 0.95 (which translates into a probability of misplacement lower than 0.05).

Subsequently, the genome draft of the isolate was compared against the reference sequences of the *Clostridium* genus, the most likely phylogenetic location. The core-genome of the *Clostridium* genus was constructed as previously described, resulting in a total of 229 core-genes associated to the *Clostridium* genus, including the isolate. A sequence concatenate of the core genome at amino acid level was built and aligned using Mafft. Core-genes were then translated to amino acids. The *Clostridium* genus phylogeny was reconstructed with IQ-tree, as previously described, using the LG + G + I model and 100 bootstrap iterations [29]. The average nucleotide identity of the core genome (cgANI) was calculated using an in-house script. In brief, the core genome of the isolate was fragmented in non-overlapping DNA sequences of 1000 bp and queried against the core-genome of all other references in that dataset. The percentage of identity was calculated for each fragment. The cgANI was then calculated as the mean identity of all fragments. We considered 2 genomic sequences to belong to the same species if the cgANI between them was higher than 95%.

We also defined the pan-genome of the *Clostridium* genus as the repertoire of gene families present in the clade. The pan-genomic reconstruction was also performed following the approach from Touchon et al. [27].

To identify possible toxins, the exotoxin database DBETH was used [30]. Pan-genome family-representative proteins were queried against DBETH using BlastP v.2.2.26. Significant hits (E-value < 0.01, coverage > 0.5) encoded by *C. cuniculi* were further characterized using BlastP against the Blast non-redundant protein database searching for specific domains and superfamily domains. Only hits that were confirmed as putative toxins using both the DBETH and the Blast nr database are shown (Additional file 4).

In addition, analysis of the *Clostridium* pan-genome, allow us to identify proteins encoded by *C. cuniculi* that are also encoded by other *Clostridium* species (i.e. presence/absence of potential virulence factors shown in Figure 4A).

### Statistical analysis

In order to determine statistically significant differences in the relative abundance of different taxa and OTUs between the group of healthy rabbits and those that developed ERE, the non-parametric Wilcoxon test was applied using wilcox.test function in the “stats” R package. Taxa and OTUs with less than 5 counts in both groups were not included in the analysis. To adjust for multiple hypothesis testing, we used the FDR approach by Benjamini and Hochberg [31] implemented in the fdr.R package. Taxa with a *p* < 0.05 and FDR < 0.1 were considered significant. First, we investigated differences between samples of healthy rabbits and those that developed ERE, collected after ERE onset. Since samples of ERE rabbits, after they developed ERE, were collected on the day 11 after weaning, on average, samples from this same day in healthy rabbits were used for comparison (results shown in Figure 1). Subsequently we studied if significant changes in bacterial taxa or OTUs between both groups of rabbits could be detected before ERE onset (Additional file 5). Accordingly, both groups were compared applying the Wilcoxon test to the relative abundance of bacterial taxa or OTUs of samples detected the day of weaning and days 1, 3, 5 and 7 after weaning. In the comparison of day 7 after weaning, samples from two ERE rabbits were not included in the analysis, since these samples corresponded to the day of ERE onset in these two particular rabbits. Besides, as a third comparison, samples obtained the morning (8–9 a.m.) of the day of ERE onset (during that particular day a drop in food consumption was detected) were compared to samples obtained from age-matched healthy littermates using the same statistical analysis (Figure 3). On average, samples collected on the day of onset in ERE rabbits correspond to the day 9 after weaning. For this reason, we compared ERE onset samples with samples from healthy rabbits obtained on day 9 after weaning.

**Figure 2:**
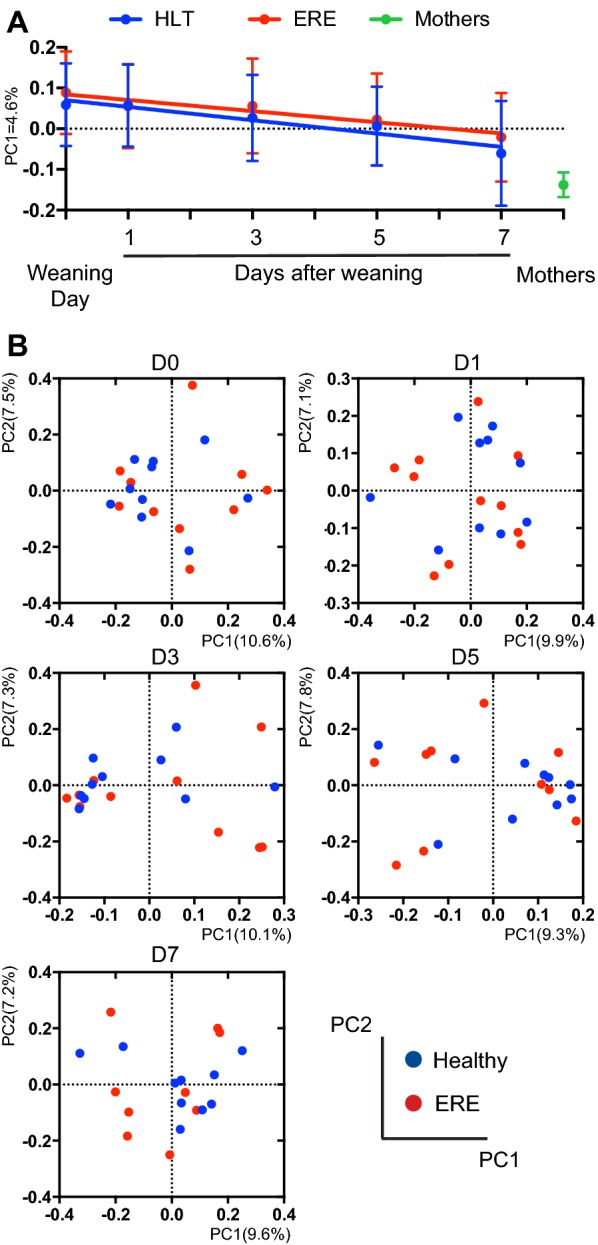
**Evolution of the microbiota after weaning and before ERE onset.** Microbiota composition before ERE onset was analyzed in caecotrophs samples collected from rabbits weaned at 30 days of age. Samples from rabbits with ERE, collected the day of the disease onset or after disease initiation, are not included in the analysis. Since most rabbits initiated ERE on day 9 or after, only the day of weaning (day 0) and days 1, 3, 5 and 7 after weaning are shown. **A** First principal coordinate of analysis of the PCoA from the unweighted UniFrac values obtained between all samples, plotted over time. Mean ± SD is shown for each time point. Samples from healthy (HLT) and ERE rabbits moved from the positive values of the axis defined by PC1 towards the negative values of the axis where the samples from the mothers are located (F test, *p* < 0.05). No significant difference between both groups in the slope of the regression line was detected (ANCOVA, *p* > 0.05). **B** PCoA at different days after weaning obtained from the unweighted UniFrac distance matrix generated between samples from a specific day. No differences were detected using the unweighted UniFrac distance at any analyzed time point (AMOVA, *p* > 0.05). *N* = 8–11 per group and time point. Two samples from four mothers, collected the day of weaning and 7 days afterwards were also included in the analysis and are shown in **A**.

For determining if the evolution of the rabbits’ microbiota since the day of weaning until day 7 post-weaning is different in healthy rabbits compared to those that will develop ERE, linear model regression analysis (Figure 2A) was performed using Graphpad.

## Results

### Epizootic rabbit enteropathy is characterized by dysbiosis of the intestinal microbiota after disease onset

To evaluate the role of dysbiosis in ERE development, we prospectively collected caecotroph samples from 33 littermate rabbits during 3 consecutive weeks, starting the day of weaning. Caecotrophs, soft feces made up of essentially caecal contents and secreted by rabbits at dawn, were collected instead of regular fecal samples since the microbiota of caecotrophs is equivalent to that one from the caecum (Additional file 6) and oral inoculation of caecal contents of sick animals has been shown to reproduce the disease in SFP rabbits [1]. Food consumption was measured daily after weaning (Additional file 3) and careful visualization of appearance of ERE compatible signs (“Materials and methods”; Additional file 2) was performed in order to detect those rabbits that develop ERE. One rabbit died immediately after weaning from a disease non-related to ERE and was excluded from the study. From the 32 rabbits available for the study, the majority (66%, *N* = 21) increased their food intake after weaning (Additional file 3) and did not develop any ERE compatible signs. In contrast, 34% of the rabbits (*N* = 11) developed a sharp decrease in food intake (> 50% reduction) during days 7 to 12 after weaning (Additional file 3). None of these 11 rabbits increased their food intake afterwards (Additional file 3) and all of them developed clinical signs compatible with ERE including diarrhea and prostration (Additional file 2). In addition, a necropsy analysis identified gross lesions compatible with ERE in all rabbits that developed a sharp decrease in food intake and ERE compatible signs (Additional file 2). As expected, no gross lesions compatible with ERE were detected in those rabbits that did not develop a sharp decrease in food intake or other signs of disease.

Previous studies have described ERE as a disease associated with microbial dysbiosis [11]. In order to confirm that dysbiosis was associated with ERE development, we analyzed, using 16S rRNA high-throughput sequencing, the microbiota composition of samples collected after ERE onset, defined as the day in which a sharp drop in food consumption was detected (see “Materials and methods” and Additional file 1). We first analyzed the overall microbial diversity by calculating the Shannon index, which takes into account the number and relative abundance of OTUs found in a given sample. This analysis showed that rabbits with ERE had lower microbiota diversity than healthy controls (lower Shannon index, *p* < 0.05, Figure 1A). Principal Coordinate of Analysis (PCoA) of unweighted UniFrac distances separated samples by health status indicating that the microbiota composition of rabbits with ERE differed from that of healthy controls (Figure 1B; AMOVA, *p* < 0.001). Similar results were obtained using the weighted UniFrac (AMOVA, *p* < 0.001). We next studied changes in specific bacterial taxa due to ERE development. At the genus level, the relative abundance of multiple genera (19 out of 50 analyzed genera) differed between rabbits with ERE and healthy controls (Wilcoxon test, *p* < 0.05, FDR < 0.1, Figure 1C). Specifically, the microbiota of rabbits with ERE had higher levels of the genera *Akkermansia*, *Escherichia/Shigella*, *Lysinibacillus*, *Campylobacter*, *Bacteroides*, *Robinsoniella*, *Sporacetigenium*, *Anaerovorax*, *Turicibacter* and *Clostridium* and lower levels of *Coprococcus*, *Olsenella*, *Slackia*, *Adlercreutzia*, *Moryella*, *Persicirhabdus*, *Subdoligranulum*, *Blautia* and *Acetivibrio*. In addition, the relative abundance of 5 phyla, 9 classes, 9 orders, 13 families and 106 OTUs differed between both groups (*p* < 0.05, FDR < 0.1) (Additional file 7). Altogether these data confirm that intestinal microbiota of rabbits that already developed ERE has a marked intestinal dysbiosis involving many different species and taxa.

### The intestinal microbiota of healthy rabbits and those that develop ERE does not differ before disease onset

In order to elucidate if the microbiota changes detected were consequence of the disease state or could be responsible for triggering ERE, we analyzed the composition of the microbiota of rabbits before the onset (from weaning until day 7 post-weaning). We first applied unweighted UniFrac distance and PCoA to analyze overall changes in the microbiota after weaning and before ERE onset. A slight change of the microbiota over time could be identified in PC1 (Figure 2A). Indeed, samples from healthy and ERE rabbits moved from the positive values of the axis towards the negative values where the samples collected from the mothers are located (F test, *p* < 0.05). Nevertheless, the identified change was similar in both healthy and ERE rabbits (ANCOVA, *p* > 0.05). In addition, when different time points were analyzed separately (Figure 2B), no separation was detected by disease state (AMOVA, *p* > 0.05). On the other hand, those bacterial genera that were altered after ERE onset (Figure 1C) evolve in abundance in a similar manner before disease onset in rabbits that will develop ERE as compared to those that remain healthy (Additional file 5). Moreover, no significant differences were detected in any taxa or OTU, at any time-point, before ERE onset (Wilcoxon test, FDR > 0.1), nor in the Shannon diversity index (Additional file 8). Altogether, these results suggest that the dybiosis that occurs in rabbits with ERE is a consequence of the disease rather than its initial cause.

### Bloom of a novel *Clostridium* species characterizes the ERE onset

**Figure 3:**
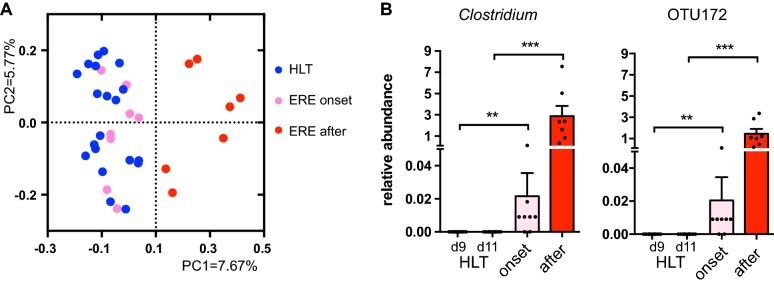
**Expansion of a single** ***Clostridium*** **OTU characterizes the microbiota of the ERE onset.** Microbiota composition was analyzed in caecotrophs samples collected from rabbits that developed ERE (the day of ERE onset and after onset) and compared with healthy littermate controls (HLT). **A** PCoA obtained from the unweighted UniFrac values calculated between samples from rabbits with ERE collected the day of onset or after onset and samples from healthy rabbits at similar time points (days 9 and 11 after weaning). The microbiota of samples collected after ERE onset was significantly different from samples of healthy controls and from samples collected the day of ERE onset (AMOVA, *p* < 0.001). However, the overall microbiota composition the day of ERE onset did not differ from that one of healthy controls (AMOVA, *p* > 0.05). **B** Relative abundance of the genus *Clostridium*, the only change detected in ERE rabbits on the day of onset compared to healthy littermates (Wilcoxon two-sided test, *p* < 0.05, FDR < 0.1). The altered abundance of this genus in ERE rabbits was due to a single OTU (OTU172). **p < 0.01,***p < 0.001. The mean ± SEM for each group is shown. *N* = 7–10 per group and time point.

ERE is an intestinal disorder that develops very rapidly (rabbits diminish food intake abruptly and other signs of the disease appear within 48 h after disease initiation [8]). It is possible that changes in the microbiota could occur very close to the appearance of the symptoms (hours before the first symptom). For this reason, we analyzed samples that were collected early in the morning the day of ERE onset, before the first symptom appeared (see “Materials and methods”). Unweighted UniFrac measure of dissimilarity and PCoA were used to compare the microbiota structure of these samples with those collected after ERE onset and from samples of age-matched healthy littermates. The microbiota from samples obtained after ERE onset differed from those collected the day of onset and from samples of healthy controls (AMOVA, *p* < 0.001; Figure 3A). However, no significant difference in the microbiota structure was detected between samples collected the day of onset and those from age-matched healthy controls (AMOVA, *p* > 0.05; Figure 3A). Similar results were obtained using the weighted UniFrac distance (AMOVA, *p* > 0.05, ERE onset vs. healthy). Although no differences were detected in the overall intestinal microbiota structure, the relative abundance of one particular taxon, the genus *Clostridium*, was found to be significantly higher in those rabbits that develop ERE (the day of ERE onset) compared to samples collected at similar time-points from healthy littermate controls (Figure 3B). Moreover, this difference was due to the presence of a single *Clostridium* OTU (OTU172) in ERE rabbits, which was absent from healthy animals (Figure 3B). Notably, this OTU was not detected in any sample collected at any time point from healthy rabbits (1 392 113 sequences, 74 samples). In contrast, *Clostridium* OTU172 was detected in samples collected the day of onset or before onset in all ERE rabbits, except for one ERE rabbit, in which this OTU was detected after disease onset.

Importantly, the *Clostridium* spp. expansion was the unique microbiota change detected the day of ERE onset. In fact, no other statistically significant differences, at any taxonomical level or in any other OTU (FDR > 0.1) or in the Shannon diversity index (Additional file 8) were identified between healthy controls and samples collected the day of ERE onset. This result demonstrates that out of 161 microbiota changes (Additional file 7) associated with ERE, only one change could be linked to the initiation of the disease.

**Figure 4:**
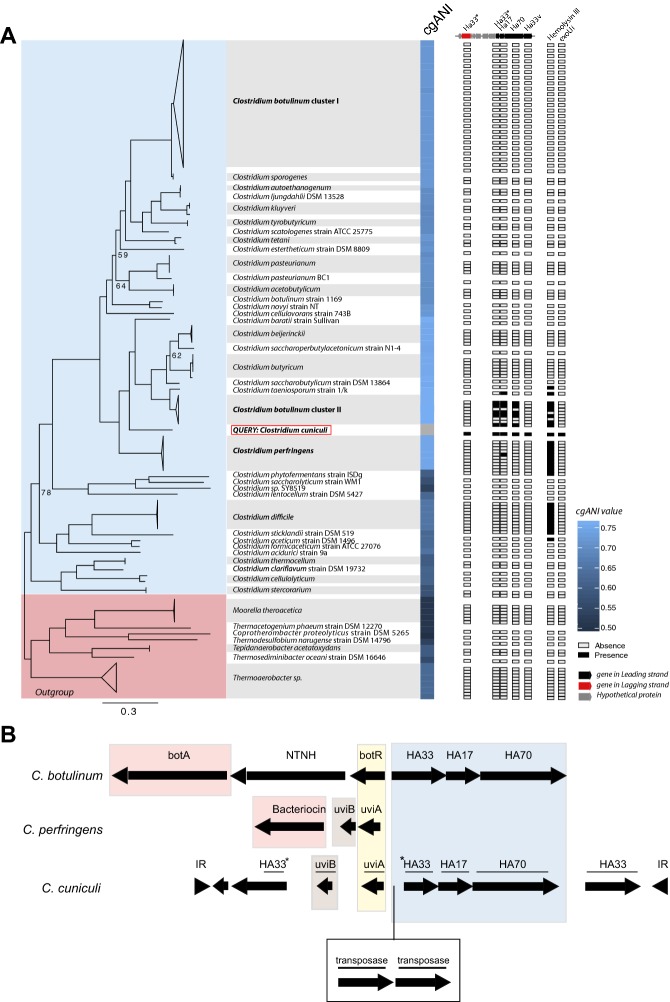
**Characterization of the OTU172 (*C. cuniculi*).** The OTU172 (unique change identified the day of ERE onset) was isolated from a caecotroph sample collected from a rabbit developing ERE. Its genome was sequenced and compared with representative sequences from the 98 genomes classified as Clostridiales within the GenBank RefSeq complete genome database (see “Materials and methods”). **A** The tree represents the best phylogenetic location of the OTU172 and species belonging to the *Clostridium* clade. The bootstrap value is stated when it was lower than 97%. The heatmap represents percentage of identity of the OTU172 with other species of the *Clostridium* clade calculated by implementation of cgANI. The analysis revealed that OTU172 represents a novel species within the genus *Clostridium,* which we named *Clostridium cuniculi* (cgANI < 95% compared to any member of the Clostridiales clade). Tree branches length represent the number of amino acid changes per site. The presence/absence in the different *Clostridium* species of potential virulence factors identified in *C. cuniculi* (discussed in the manuscript) is indicated. This include an operon shown in more detail in **B**, a hemolysin and a gene similar to the cytotoxin exoU. **B** The schematic representation of the operon detected in the genome of *C. cuniculi* and corresponding operons in genomes of *C. botulinum A str. ATCC 3502* and *C. perfringens* SM101, plasmid pSM101B. The names above *C. cuniculi* ORFs correspond to the best matches against ORFs present in the other operons (E < 1e−15). ORFs that did not match the *C. botulinum* or *C. perfringens* operons were blast against the NCBI nr database. Its best match is indicated (E < 1e−101). If the coverage against the reference ORF was lower than 66%, the ORF is marked with an asterisk. Only ORFs with > 60 amino acids are shown. In yellow are indicated transcriptional regulators; in red, effector proteins; in blue, hemagglutinins with a similar organization in *C. cuniculi* and *C. botulinum*, and in brown putative holins. *botA* botulinum toxin A, *NTNH* nontoxic, nonhemagglutinin protein, *HA* hemagglutinin, *IR* inverse repeats.

Subsequently, we isolated the *Clostridium* OTU172 (see “Materials and methods”) and tried to reproduce the disease by orally inoculating the obtained isolate into SPF rabbits. However, we were not able to reproduce the symptoms of the disease, neither with the caecal contents from sick animals, which previous studies demonstrated to be sufficient to induce ERE in rabbits [1]. These results suggested that either the SPF rabbits used in our study were more resistant to the disease or either the inoculum dose utilized was not sufficient to induce the disease. Nevertheless, in order to get an insight into the potential role of *Clostridium* OTU172 in the initiation of ERE, we sequenced the genome of the obtained isolate. We first compared the genome of this OTU with all the genomes (*N* = 98) of members of the Clostridiales order deposited in the GenBank RefSeq database. Notably, this analysis identified the *Clostridium* OTU172 as a novel species within the genus *Clostridium* [core genome average nucleotide identity—(cgANI) < 95% compared to any member of the Clostridiales clade]. We named this novel species as *Clostridium cuniculi*, considering the genitive Latin name of the host from which it was isolated. Interestingly, *C. cuniculi* is phylogenetically related to the two well-characterized pathogens *C. perfringens* and *C. botulinum* (Figure 4A). In addition, the analysis of the open reading frames (ORFs) encoded by *C. cuniculi* identified several putative toxins by which *C. cuniculi* could influence ERE development (Additional file 4). The identified ORFs include a protein similar to exoU (a type III effector protein with cytotoxic activity) and a protein similar to the hemolysin type III. In addition, an operon strikingly similar to the operon present in *C. botulinum*, which encodes the type A neurotoxin complex responsible for the botulism disease, was also identified in the genome of *C. cuniculi* (Figure 4B). The operon identified in *C. cuniculi* encodes all the hemagglutinin components of the type A neurotoxin complex, although it lacks the effector botA protein responsible for the muscle paralysis, characteristic of botulism. Interestingly, the type A neurotoxin complex, lacking botA induces the destruction of the intestinal villi [32], a phenomenon that has also been detected in rabbits suffering ERE [33]. Notably, all these potential virulence factors identified in *C. cuniculi* are exclusively or almost exclusively encoded by pathogenic *Clostridium* species (i.e. *C. botulinum*, *C. perfringens* and *C. difficile*, Figure 4A).

Altogether, our results indicate that the dysbiosis detected after the initiation of the pathology is a consequence rather the initial cause of the disease and pinpoint *C. cuniculi* as a potential candidate involved in the initiation of ERE.

## Discussion

Here, we have applied 16S rRNA high-throughput sequencing in order to evaluate changes in the intestinal microbiota through the complete progression of ERE, one of the most devastating diseases in rabbits. We first characterized the microbiota of rabbits after ERE initiation and compared it with age-matched littermate controls. This first analysis identified major changes in the microbiota of rabbits that had developed ERE. This result is consistent with a previous study [11], in which the caecal microbiota of rabbits that had develop ERE (after onset) was also characterized using 16S rRNA high-throughput sequencing. Consistent with our analysis, the authors also identified a pronounced dysbiosis in samples from the caecum of ERE rabbits as compared to healthy controls. Similar to our study, the authors showed that rabbits that develop ERE harbor a microbiota with lower diversity and altered abundances of several bacterial taxa, including higher levels of *Akkermansia*, *Bacteroides* and *Clostridium*. In contrast to our study, the authors did not analyze the composition of the microbiota of rabbits before onset. For this reason, the authors discussed that the detected dysbiosis could be a consequence of the disease instead of its initial cause, which we have demonstrated by applying a longitudinal prospective study design. Although not involved in disease initiation, the microbiota changes detected after onset may play a role in the progression of the disease. For example, an expansion of *Campylobacter* spp. was detected, after disease onset, in most of the rabbits that developed ERE. Species belonging to this bacterial genus represent one of the most frequent pathogens causing diarrhea [34], a sign that is frequently detected in rabbits that develop ERE.

Although major dysbiosis is not the initial cause of ERE, a single change in the microbiota composition could be detected in caecotroph samples early in the morning of the ERE onset day. This microbial change does not seem to be the consequence of ERE since the sample was collected immediately before the reduction in food consumption (the first sign of ERE). This bacterial change consists on the expansion of a single *Clostridium* species. A previous study using culturing and PCR typing methods identified higher counts of *Clostridium perfringens* in samples collected from ERE rabbits after disease onset [1]. However, a role for *C. perfringens* in ERE development has never been proved. We were able to isolate and sequence the genome of the *Clostridium* species that expanded in rabbits the day of ERE onset. Interestingly, this bacterial species was phylogenetically related to *C. perfringens*. Nevertheless, by comparing its genome with 98 Clostridiales genomes, including several genomes of *C. perfringens*, we were able to elucidate that the *Clostridium* species that could be linked to the initiation of ERE is a novel bacterial species, different from *C. perfringens*, and we named it *C. cuniculi*.

*Clostridium cuniculi* encodes several putative toxins by which it could potentially influence ERE development. One of these toxins is similar to exoU, a cytotoxin encoded by *Pseudomonas aeruginosa* that causes irreversible damage of mammalian cells and cell death [35]. Notably, higher apoptosis of cells on the jejunum epithelium has been detected in rabbits developing ERE signs [33]. On the other hand, *C. cuniculi* also encodes for an operon that is very similar to the operon present in *C. botulinum*, which encodes the type A neurotoxin complex, responsible for the botulism disease. The operon identified in *C. cuniculi* encodes the three hemagglutinin components of the type A neurotoxin complex, which are transcribed from the same DNA strand (Figure 4B). In the other DNA strand, the *C. botulinum* operon encodes in this order: botR, non-toxin non-hemagglutinin protein (NTNH) and botA. botR is a transcriptional regulator that is known to induce the expression of botA, the effector protein of the complex, that inhibits the release of the neurotransmitter acetylcholine from peripheral cholinergic synapses, inducing muscle paralysis, characteristic of botulism [36, 37]. Following a similar organization, in the strand opposite to the one that encodes the hemagglutinins, the *C. cuniculi* operon also encodes for a transcriptional regulator (uviA). This regulator is known to induce the expression of a bacteriocin, the effector protein of the operon in *C. perfringens*, that is encoded downstream of uviA [36]. In addition, downstream of uviA, *C. perfringens* encodes uviB, a protein that presumably enhances the secretion of the bacteriocin [38]. *C. cuniculi* also encodes for uviB, however, in the place of the effector protein, the *C. cuniculi* operon encodes a protein partially similar to one of the hemagglutinins (H33). Interestingly, Interproscan analysis of this protein identified one domain that is similar to the I66 domain (*p* < 1e−15). This domain is present in serine protease inhibitors. Acetylcholinesterase is a serine protease that catalyzes the breakdown of acetylcholine, and its inhibition could lead to paralysis. Notably, intestinal paralysis is thought to be the cause of some of the ERE signs, such as caecal impaction. Thus this particular protein, may be promoting some of the ERE signs through interference with the cholinergic synapse. Future studies should be performed in order to demonstrate this hypothesis and confirm the role of this protein in the development of ERE. Besides the possible role of this particular protein in ERE development, the whole operon could also play a role in the development of ERE. Indeed, a truncated type A neurotoxin complex from *C. botulinum*, not containing the effector protein botA (such as the one found in *C. cuniculi*), has been shown in vitro to induce formation of vacuoles in the cytoplasm of intestinal epithelial cells, resulting in cell death [32]. Moreover, this “non-toxic” type A complex promotes the destruction of the mucosal surface of the intestinal villi in rats. Consistent with this, the intestinal villi from rabbits that develop ERE are atrophied and contain a higher number of apoptotic enterocytes, some of which display cytoplasm vacuolization [33]. Therefore, future studies should elucidate the role of the proteins encoded by the *C. cuniculi* operon in the intestinal damage detected in ERE.

Taking into account our results and previous studies showing that ERE can be transmitted by oral inoculation of intestinal contents from dead animals, not containing detectable virus or parasites [1, 7, 8], a bacterial infection, possibly by *C. cuniculi,* seems to be the most likely cause of ERE. We tried to reproduce the disease by inoculating *C. cuniculi* to SPF rabbits; however, we were not able to reproduce the symptoms of the disease even with the caecal contents from sick animals, used as positive control. This result suggests that the SPF animals utilized in our study, might be more resistant to the disease than those used by Marlier et al. [1]. Moreover, it is possible that the inoculum dose used in our experiments was not sufficient to reproduce the symptoms of the disease. In addition, it is possible that the in vitro cultivation of *C. cuniculi* diminishes its infectivity potential, or that other strains, different from the isolated one, have a higher virulent potential. Lastly, other bacteria, besides *C. cuniculi*, may be required for the development of ERE symptoms. Further efforts should be performed in order to confirm the role of *C. cuniculi* in ERE development.

In any case, an infectious process is consistent with the results obtained in our study. In the first stages of the infectious process, the pathogen could represent a very small fraction of the whole bacterial populations residing in the intestinal tract, thus the microbiota structure of healthy and sick rabbits could be equivalent before the initiation of ERE. Indeed, the morning of the ERE onset day, *C. cuniculi* represented a very minor population within the microbiota (average 0.02%) and the microbiota structure was still not altered. Subsequently, pathological disturbances, consequence of the infectious process could have an effect on the overall microbiota composition and lead to the high number of bacterial changes detected in rabbits with ERE. For example, as mentioned before, the first sign that occurs in ERE is a sharp decrease in food consumption, and it has been shown that food restriction can cause microbiota changes in rats [39]. It is therefore plausible that some of the changes identified in the microbiota of ERE rabbits could have been influenced by the decrease in food consumption. In fact, one of the changes detected after disease onset (increase in the abundance of the genus *Anaerovorax* in ERE rabbits) could be detected in rabbits restricted to ingest an amount of food similar to that ingested by ERE rabbits (Additional file 9). Nevertheless, the majority of the bacterial changes detected during ERE, including the expansion of *Clostridium* spp. were not induced by restriction in food consumption (Additional file 9). In addition, restricted food consumption per se did not promote ERE signs.

In summary, we have characterized for the first time, the changes in the microbiota composition of rabbits before, during and after ERE onset. Our results have confirmed that ERE is associated with a pronounced dysbiosis of the bacterial communities residing in the large intestinal tract. However, by investigating the microbiota composition before the development of the ERE symptoms, we found that only 1 out of 161 bacterial changes associated with ERE could be linked to the initiation of the disease. The single identified change was the gut colonization by a novel *Clostridium* species, exclusively detected in rabbits that developed ERE. The identified species encodes several putative toxins and it is phylogenetically related to the two well-characterized pathogens *C. botulinum* and *C. perfringens*, suggesting an infectious origin for ERE rather than a dysbiosis related origin.

## Additional files

**Additional file 1.** **Changes in food consumption in ERE and healthy rabbits.** Fold change in food consumption (grams of food consumed by a particular rabbit during 24 h divided by grams of food consumed by that rabbit during previous 24 h). Fold changes in food consumption from healthy rabbits during the first 2 weeks post-weaning (blue dots), ERE rabbits before disease onset (green dots) or ERE rabbits the day of ERE onset (pink dots) are shown. The mean for each group ± SEM is also shown. The day of ERE onset is considered as the day in which a fold change lower than 0.5 was detected. All ERE rabbits developed other ERE compatible signs following the drop in food consumption (see “Materials and methods”, Additional file 2). A fold change of 0.5 was used since a few healthy rabbits diminished food intake, but to a lower extent, on specific days, without developing any sign consistent with ERE. Two sided T-test, ****p < 0.0001, ns: not significant. *N* = 11-21 rabbits per group.

**Additional file 2.** **Signs and gross lesions identified in ERE rabbits.** In order to confirm ERE development, a necropsy was performed in all rabbits by a veterinarian specialist in anatomical pathology (co-author Jorge Martínez). Gross lesions compatible with ERE, such as distension of the stomach and intestine, caecum filled with gas, liquid or impacted with solid/dry content, and presence of abundant quantities of mucus in the colon were observed in all rabbits that developed a sharp decrease in food confirming the development of ERE. P = presence of a particular sign/gross lesion. ^a^Rabbits are sorted in the same order as ERE rabbits shown in Additional file 3. Healthy rabbits are not shown since they did not develop any of the signs/gross lesions.

**Additional file 3.** **Food intake by rabbits after weaning.** The amount of food (g) consumed by rabbits on different days after weaning is shown as a color-coded heatmap. Day 0 = day of weaning. NA: not analyzed due to death or euthanasia of that particular rabbit. The day of ERE onset as revealed by a sharp drop in food intake (> 50% reduction compared to the previous day) is indicated with an asterisk. Healthy rabbits (HLT) were followed for another week. No signs of disease were detected in HLT rabbits during this additional week. *N* = 32 (21 HLT and 11 ERE rabbits).

**Additional file 4.** **Putative endotoxins identified in** ***C. cuniculi*. Isolate-specific proteins were queried against DBETH database in order to identify potential exotoxins.** Significant hits (E-value < 0.01, coverage > 0.5) were further characterized using BlastP against the Blast non-redundant protein database searching for specific domains and superfamily domains. Only hits that were confirmed as putative toxins using both the DBETH and the Blast database are shown.

**Additional file 5.** **Equivalent bacterial genera abundance in healthy and ERE rabbits before onset.** Microbiota composition before ERE onset was analyzed in caecotrophs samples collected from rabbits weaned at 30 days of age. Samples from rabbits with ERE, collected the day of the disease onset or after disease initiation, are not included in the analysis. Since most rabbits initiated ERE on day 9 or after, only the day of weaning (day 0) and days 1, 3, 5 and 7 after weaning are shown. Each panel shows the relative abundance, at different days after weaning of a bacterial genus that was found to be significantly different in rabbits that had developed ERE (Figure 1C). Horizontal lines represent the mean for each group. *N* = 8–11 per group and time point. No significant differences between healthy (HLT) and ERE rabbits were detected in any taxonomic level or OTU, at any time-point, before ERE onset (Wilcoxon two-sided test, FDR > 0.1).

**Additional file 6.** **Caecal and caecotroph microbiota of rabbits.** Phylogenetic classification of 16S rDNA frequencies in the caecum or caecotroph samples from healthy rabbits, 21 days after weaning. Each bar represents the microbiota of an individual rabbit whose ID number is indicated above the bar. The most predominant bacterial taxa are shown and labeled with different colors as indicated. Bacterial taxa were obtained by classification of 16S rDNA sequences to the genus level using Mothur. In case a sequence could not be classified to the genus level, the closest level of classification to the genus level was given, preceded by “unclassified_”. *N* = 6.

**Additional file 7.** **Statistically significant differences between rabbits with ERE (after disease onset) and age-matched healthy littermate controls.** The microbiota composition of samples collected after ERE onset showed significant differences in abundances of hundreds of bacterial groups compared to the microbiota composition of healthy littermate controls (Wilcoxon test, *p* < 0.05, FDR < 0.1).

**Additional file 8.** **Microbial diversity of rabbits before onset and the day of onset.** (A) Shannon diversity index of caecotroph samples from rabbits at specific days after weaning and before ERE onset. D0 = day of weaning. Samples from rabbits with ERE obtained on the day of onset or after disease initiation are not included in the analysis. Since most rabbits initiated ERE on day 9 or after, only days 0, 1, 3, 5 and 7 after weaning are shown. As control, the Shannon index of healthy (HLT) littermate rabbits is shown at similar time points. (B) Shannon diversity index from samples obtained from sick rabbits the day of onset compared with samples from HLT littermate controls at a similar time point (day 9 after weaning). Horizontal lines represent the mean for each group. *N* = 8–11 per group and time point. No significant differences were detected in the Shannon index between ERE and healthy rabbits at any time point analyzed (Wilcoxon two-sided test, *p* > 0.05).

**Additional file 9.** **The majority of the changes detected after the initiation of ERE are not due to a decrease in food intake.** Relative abundance of the genus *Anaerovorax* or the genus *Clostridium* in samples collected from 6 weeks old healthy rabbits that were forced to consume an amount of food similar to that of ERE rabbits during 48 h (food restricted) as compared to healthy age-match controls receiving food ad libitum. A caecotroph sample was collected from each rabbit and its microbiota composition was analyzed. From all changes detected in ERE rabbits, only an increase in the genus *Anaerovorax* was found to occur after food restriction, similarly to what was observed in ERE rabbits after the disease onset (Wilcoxon two-sided test, FDR < 0.1). Food restriction did not cause the expansion of *Clostridium* spp. detected in ERE rabbits. ***p < 0.001. *N* = 6–7 per group.

## Abbreviations

ERE: epizootic rabbit enteropathy
SPF: specific pathogen free
PVC: polyvinyl chloride
OTU: operational taxonomic unit
AGC: abundance based greedy clustering
dNTP: deoxynucleotide triphosphate
ORF: open reading frame
cgANI: core genome average nucleotide identity
PCoA: principal coordinate of analysis
FDR: false discovery rate
HA: hemagglutinin
botA: botulinum toxin A
NTNH: non-toxin non-hemagglutinin protein
