# Supplementary figures and images for: Gut colonization by a novel Clostridium species is associated with the onset of epizootic rabbit enteropathy
Source: Vet Res. 2018 Dec 20;49:123. doi: 10.1186/s13567-018-0617-8 (PMC6302431; doi:10.1186/s13567-018-0617-8)

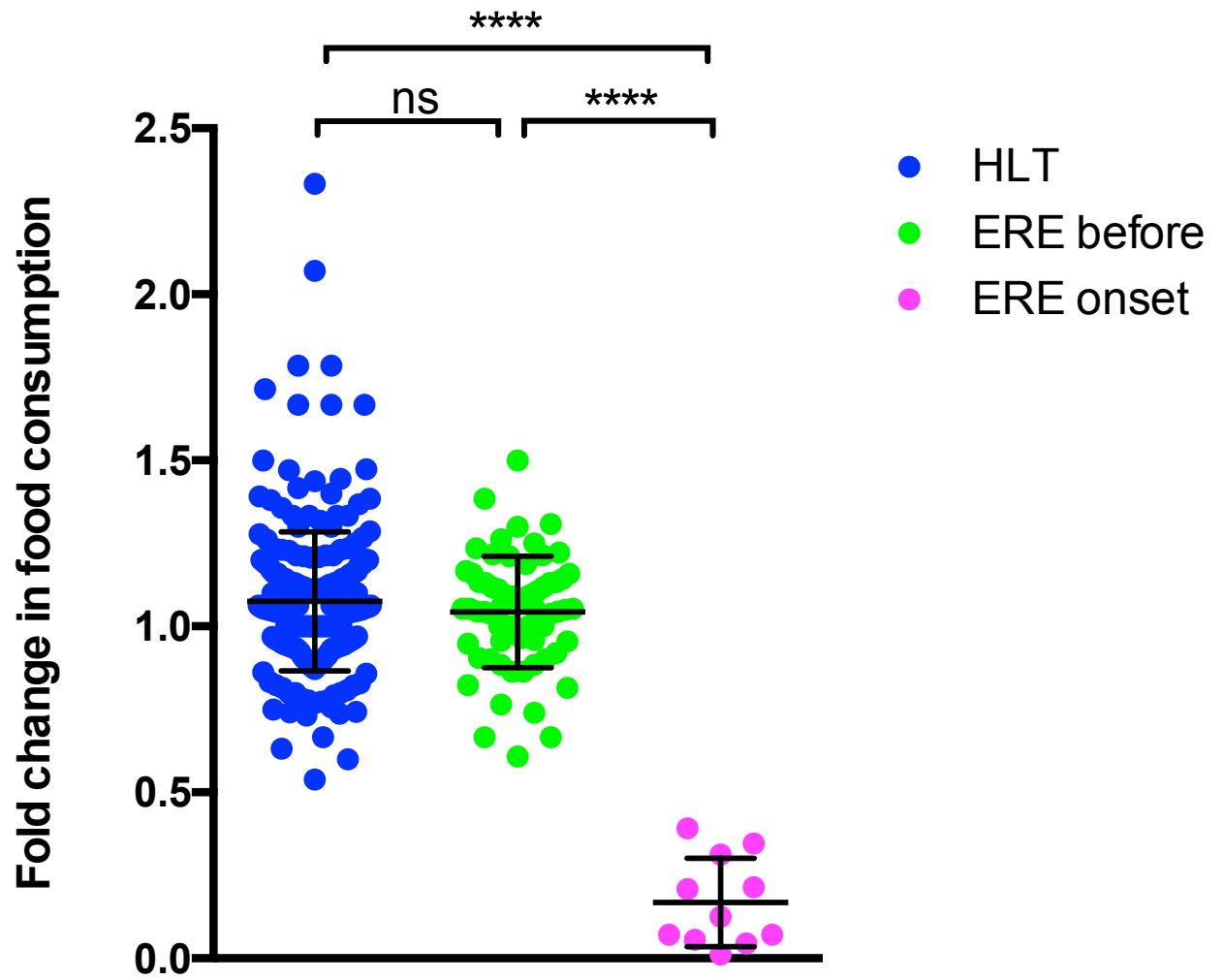

Supplement: Supplementary file 1 — Additional file 1. Changes in food consumption in ERE and healthy rabbits. Fold change in food consumption (grams of food consumed by a particular rabbit during 24 h divided by grams of food consumed by that rabbit during previous 24 h). Fold changes in food consumption from healthy rabbits during the first 2 weeks post-weaning (blue dots), ERE rabbits before disease onset (green dots) or ERE rabbits the day of ERE onset (pink dots) are shown. The mean for each group ± SEM is also shown. The day of ERE onset is considered as the day in which a fold change lower than 0.5 was detected. All ERE rabbits developed other ERE compatible signs following the drop in food consumption (see “Materials and methods”, Additional file 2). A fold change of 0.5 was used since a few healthy rabbits diminished food intake, but to a lower extent, on specific days, without developing any sign consistent with ERE. Two sided T-test, ****p < 0.0001, ns: not significant. N = 11-21 rabbits per group. [file 13567_2018_617_MOESM1_ESM.pdf]

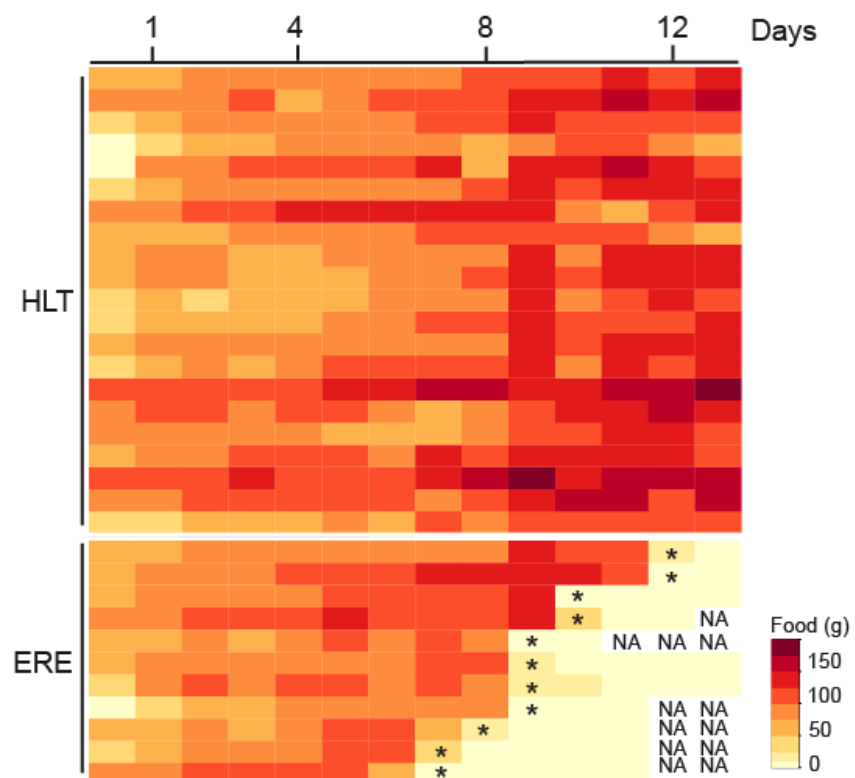

Supplement: Supplementary file 3 — Additional file 3. Food intake by rabbits after weaning. The amount of food (g) consumed by rabbits on different days after weaning is shown as a color-coded heatmap. Day 0 = day of weaning. NA: not analyzed due to death or euthanasia of that particular rabbit. The day of ERE onset as revealed by a sharp drop in food intake (> 50% reduction compared to the previous day) is indicated with an asterisk. Healthy rabbits (HLT) were followed for another week. No signs of disease were detected in HLT rabbits during this additional week. N = 32 (21 HLT and 11 ERE rabbits). [file 13567_2018_617_MOESM3_ESM.pdf]

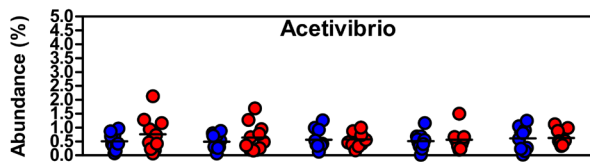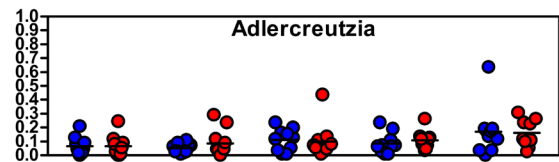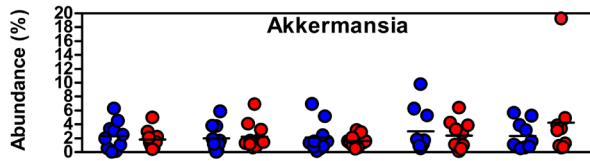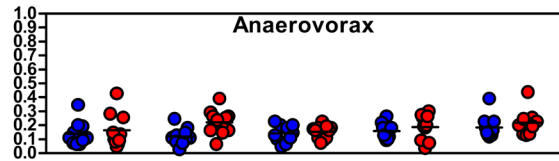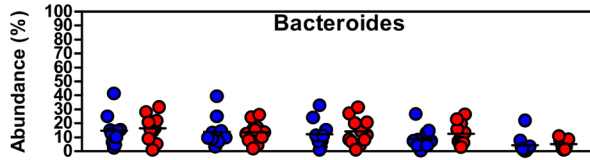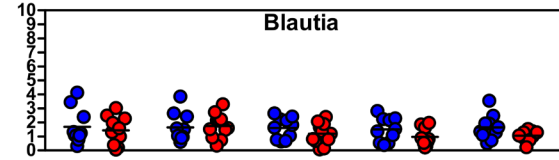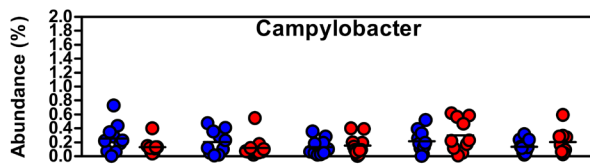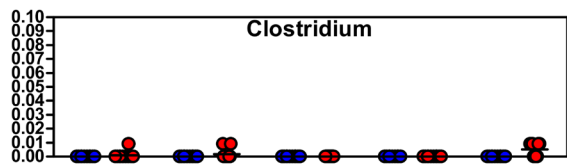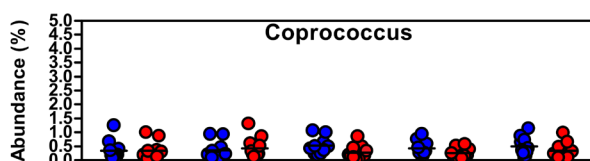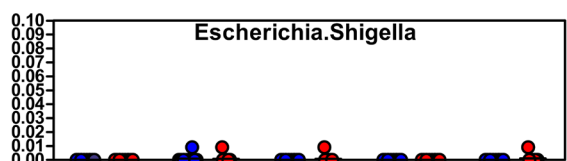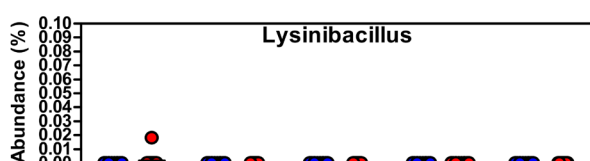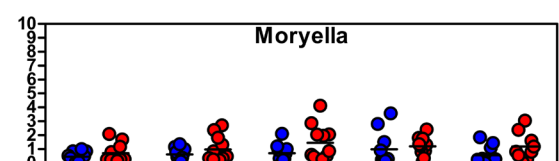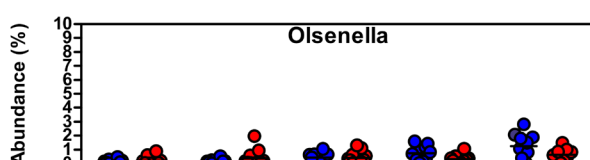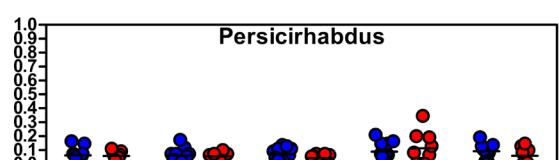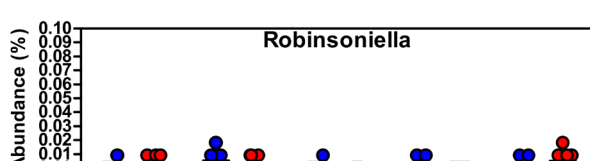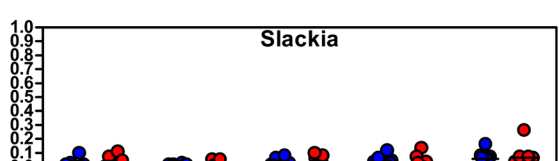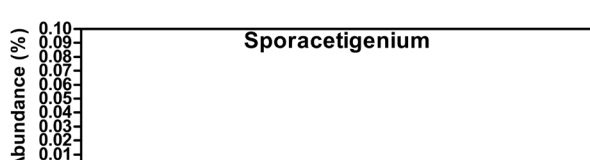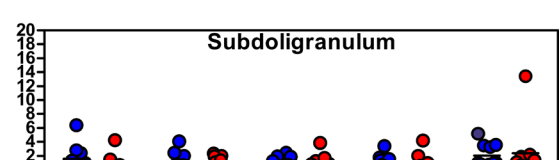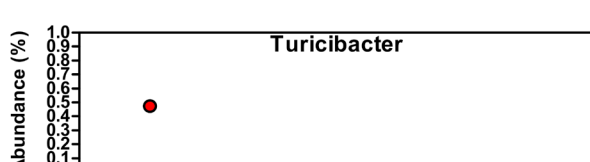

● HLT ● ERE

Supplement: Supplementary file 5 — Additional file 5. Equivalent bacterial genera abundance in healthy and ERE rabbits before onset. Microbiota composition before ERE onset was analyzed in caecotrophs samples collected from rabbits weaned at 30 days of age. Samples from rabbits with ERE, collected the day of the disease onset or after disease initiation, are not included in the analysis. Since most rabbits initiated ERE on day 9 or after, only the day of weaning (day 0) and days 1, 3, 5 and 7 after weaning are shown. Each panel shows the relative abundance, at different days after weaning of a bacterial genus that was found to be significantly different in rabbits that had developed ERE (Figure 1C). Horizontal lines represent the mean for each group. N = 8–11 per group and time point. No significant differences between healthy (HLT) and ERE rabbits were detected in any taxonomic level or OTU, at any time-point, before ERE onset (Wilcoxon two-sided test, FDR > 0.1). [file 13567_2018_617_MOESM5_ESM.pdf]

**A**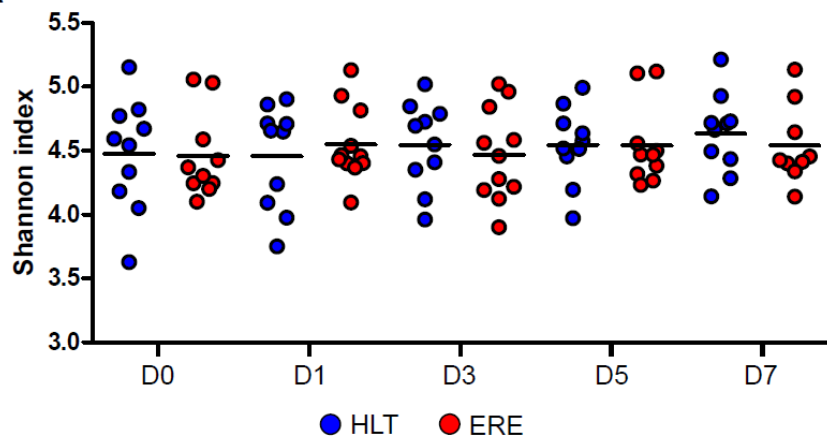**B**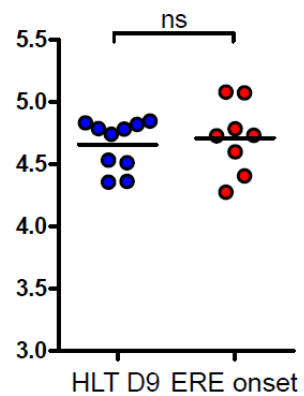

Supplement: Supplementary file 8 — Additional file 8. Microbial diversity of rabbits before onset and the day of onset. (A) Shannon diversity index of caecotroph samples from rabbits at specific days after weaning and before ERE onset. D0 = day of weaning. Samples from rabbits with ERE obtained on the day of onset or after disease initiation are not included in the analysis. Since most rabbits initiated ERE on day 9 or after, only days 0, 1, 3, 5 and 7 after weaning are shown. As control, the Shannon index of healthy (HLT) littermate rabbits is shown at similar time points. (B) Shannon diversity index from samples obtained from sick rabbits the day of onset compared with samples from HLT littermate controls at a similar time point (day 9 after weaning). Horizontal lines represent the mean for each group. N = 8–11 per group and time point. No significant differences were detected in the Shannon index between ERE and healthy rabbits at any time point analyzed (Wilcoxon two-sided test, p > 0.05). [file 13567_2018_617_MOESM8_ESM.pdf]

*Anaerovorax* spp.

*Clostridium* spp.

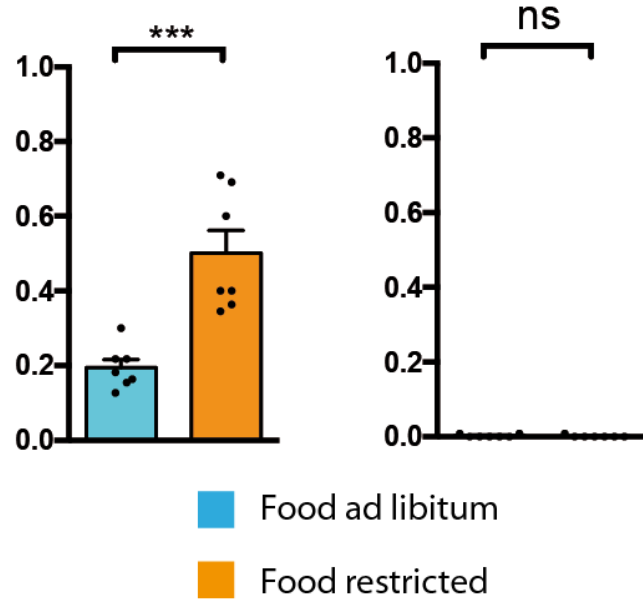

Supplement: Supplementary file 9 — Additional file 9. The majority of the changes detected after the initiation of ERE are not due to a decrease in food intake. Relative abundance of the genus Anaerovorax or the genus Clostridium in samples collected from 6 weeks old healthy rabbits that were forced to consume an amount of food similar to that of ERE rabbits during 48 h (food restricted) as compared to healthy age-match controls receiving food ad libitum. A caecotroph sample was collected from each rabbit and its microbiota composition was analyzed. From all changes detected in ERE rabbits, only an increase in the genus Anaerovorax was found to occur after food restriction, similarly to what was observed in ERE rabbits after the disease onset (Wilcoxon two-sided test, FDR < 0.1). Food restriction did not cause the expansion of Clostridium spp. detected in ERE rabbits. ***p < 0.001. N = 6–7 per group. [file 13567_2018_617_MOESM9_ESM.pdf]
