# Supplementary material for: Gut colonization by a novel Clostridium species is associated with the onset of epizootic rabbit enteropathy
Source: Vet Res. 2018 Dec 20;49:123. doi: 10.1186/s13567-018-0617-8 (PMC6302431; doi:10.1186/s13567-018-0617-8)
Supplement: Supplementary file 2 — Additional file 2. Signs and gross lesions identified in ERE rabbits. In order to confirm ERE development, a necropsy was performed in all rabbits by a veterinarian specialist in anatomical pathology (co-author Jorge Martínez). Gross lesions compatible with ERE, such as distension of the stomach and intestine, caecum filled with gas, liquid or impacted with solid/dry content, and presence of abundant quantities of mucus in the colon were observed in all rabbits that developed a sharp decrease in food confirming the development of ERE. P = presence of a particular sign/gross lesion. aRabbits are sorted in the same order as ERE rabbits shown in Additional file 3. Healthy rabbits are not shown since they did not develop any of the signs/gross lesions. [file 13567_2018_617_MOESM2_ESM.docx]

| Rabbit ID^a^ | Prostration | Diarrhea | Mucus in colon | Stomach/small intestine filled with gas/liquid | Caecal Impaction | Caecum filled with gas/liquid |
| --- | --- | --- | --- | --- | --- | --- |
| R4 | P | P |  | P | P | P |
| R7 | P | P |  | P | P |  |
| R11 | P | P | P | P |  |  |
| R15 | P | P | P | P |  |  |
| R16 | P | P |  | P |  | P |
| R18 | P | P | P | P | P | P |
| R8 | P | P |  | P | P |  |
| R24 | P | P | P | P | P | P |
| R25 | P |  | P | P |  | P |
| R27 | P | P | P | P |  | P |
| R21 | P | P |  | P | P | P |
