# Supplementary material for: Gut colonization by a novel Clostridium species is associated with the onset of epizootic rabbit enteropathy
Source: Vet Res. 2018 Dec 20;49:123. doi: 10.1186/s13567-018-0617-8 (PMC6302431; doi:10.1186/s13567-018-0617-8)
Supplement: Supplementary file 4 — Additional file 4. Putative endotoxins identified in C. cuniculi. Isolate-specific proteins were queried against DBETH database in order to identify potential exotoxins. Significant hits (E-value < 0.01, coverage > 0.5) were further characterized using BlastP against the Blast non-redundant protein database searching for specific domains and superfamily domains. Only hits that were confirmed as putative toxins using both the DBETH and the Blast database are shown. [file 13567_2018_617_MOESM4_ESM.docx]

|  | **DBETH database** | | | **Blast non redundant protein database** | | | | | |
| --- | --- | --- | --- | --- | --- | --- | --- | --- | --- |
| **GeneID *C.cuniculi*** | **Reference** | **E-value** | **Description** | **Blast specific domain** | **E-value** | **Description** | **Blast superfamily domain** | **E-value** | **Description** |
| CLCU001.0001.d01_001280 | triO34208\|O34208_PSAE | 9E-07 | ExoU Type 3 secretion effector protein | ExoU like protein domain | 7.74E-63 | Phospholipase toxin | RssA superfamily | 4.96E-35 | Phospholipase |
| CLCU001.0001.d01_028190 | triQ897Y4\|Q897Y4_CLOTE | 8E-31 | Hemolysin III | HlyIII | 1.27E-52 | Channel protein, hemolysin III family | HlyIII superfamily | 7.62E-27 | Channel protein, hemolysin III family |
