# Supplementary material for: Gut colonization by a novel Clostridium species is associated with the onset of epizootic rabbit enteropathy
Source: Vet Res. 2018 Dec 20;49:123. doi: 10.1186/s13567-018-0617-8 (PMC6302431; doi:10.1186/s13567-018-0617-8)
Supplement: Supplementary file 6 — Additional file 6. Caecal and caecotroph microbiota of rabbits. Phylogenetic classification of 16S rDNA frequencies in the caecum or caecotroph samples from healthy rabbits, 21 days after weaning. Each bar represents the microbiota of an individual rabbit whose ID number is indicated above the bar. The most predominant bacterial taxa are shown and labeled with different colors as indicated. Bacterial taxa were obtained by classification of 16S rDNA sequences to the genus level using Mothur. In case a sequence could not be classified to the genus level, the closest level of classification to the genus level was given, preceded by “unclassified_”. N = 6. [file 13567_2018_617_MOESM6_ESM.pdf]

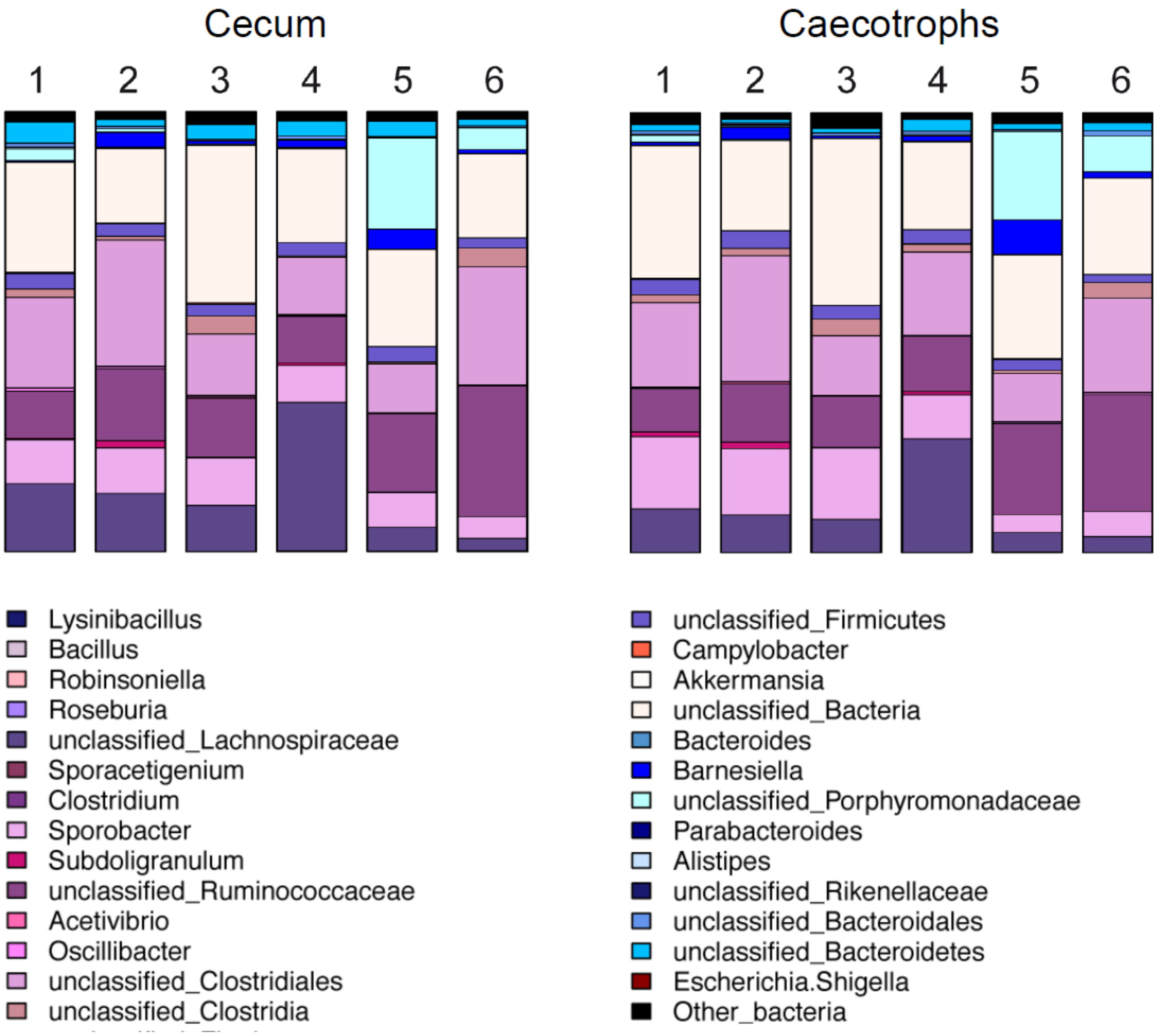
Cecum
Caecotrophs
1
2
3
4
5
6
1
2
3
4
5
6
Lysinibacillus
Bacillus
Robinsoniella
Roseburia
unclassified_Lachnospiraceae
Sporacetigenium
Clostridium
Sporobacter
Subdoligranulum
unclassified_Ruminococcaceae
Acetivibrio
Oscillibacter
unclassified_Clostridiales
unclassified_Clostridia
unclassified_Firmicutes
Campylobacter
Akkermansia
unclassified_Bacteria
Bacteroides
Barnesiella
unclassified_Porphyromonadaceae
Parabacteroides
Alistipes
unclassified_Rikenellaceae
unclassified_Bacteroidales
unclassified_Bacteroidetes
Escherichia.Shigella
Other_bacteria
