# Supplementary material for: Gut colonization by a novel Clostridium species is associated with the onset of epizootic rabbit enteropathy
Source: Vet Res. 2018 Dec 20;49:123. doi: 10.1186/s13567-018-0617-8 (PMC6302431; doi:10.1186/s13567-018-0617-8)
Supplement: Supplementary file 7 — Additional file 7. Statistically significant differences between rabbits with ERE (after disease onset) and age-matched healthy littermate controls. The microbiota composition of samples collected after ERE onset showed significant differences in abundances of hundreds of bacterial groups compared to the microbiota composition of healthy littermate controls (Wilcoxon test, p < 0.05, FDR < 0.1). [file 13567_2018_617_MOESM7_ESM.docx]

| Taxa or OTU | Fold change ERE to HLT | Wilcoxon test *p* value | FDR |
| --- | --- | --- | --- |
| **Phylum** | | |  |
| Proteobacteria | 18.8513 | 0.0015 | 0.0120 |
| Actinobacteria | 0.2290 | 0.0031 | 0.0123 |
| Firmicutes | 0.7316 | 0.0097 | 0.0213 |
| TM7 | 0.1012 | 0.0106 | 0.0213 |
| Bacteroidetes | 1.5971 | 0.0330 | 0.0440 |
| **Class** | | |  |
| Alphaproteobacteria | 67.6871 | 0.0010 | 0.0193 |
| Epsilonoproteobacteria | 12.4762 | 0.0021 | 0.0195 |
| Actinobacteria | 0.2290 | 0.0031 | 0.0195 |
| Clostridia | 0.6758 | 0.0046 | 0.0220 |
| Erysipelotrichi | 4.5515 | 0.0072 | 0.0275 |
| TM7 incertae sedis | 0.1012 | 0.0106 | 0.0337 |
| Bacilli | 96.3975 | 0.0214 | 0.0507 |
| Bacteroidia | 1.5678 | 0.0330 | 0.0570 |
| Gammaproteobacteria | 183.1933 | 0.0595 | 0.0870 |
| **Order** | | |  |
| Alphaproteobacteria incertae sedis | 148.4874 | 0.0006 | 0.0146 |
| Campylobacterales | 12.4762 | 0.0021 | 0.0267 |
| Coriobacteriales | 0.2296 | 0.0031 | 0.0267 |
| Clostridiales | 0.6760 | 0.0046 | 0.0301 |
| Bacillales | 95.3061 | 0.0069 | 0.0304 |
| Erysipelotrichales | 4.5515 | 0.0072 | 0.0304 |
| Desulfovibrionales | 5.9524 | 0.0184 | 0.0438 |
| Enterobacteriales | 194.6429 | 0.0293 | 0.0552 |
| Bacteroidales | 1.5678 | 0.0330 | 0.0572 |
| **Family** | | |  |
| Bacteroidaceae | 10.9711 | 0.0001 | 0.0035 |
| Clostridiaceae | 335.2857 | 0.0002 | 0.0035 |
| Ruminococcaceae | 0.5136 | 0.0007 | 0.0054 |
| Bacillaceae | 128.5714 | 0.0007 | 0.0054 |
| Peptostreptococcaceae | 141.2857 | 0.0008 | 0.0054 |
| Clostridiales incertae sedis XIII | 5.3886 | 0.0011 | 0.0066 |
| Campylobacteraceae | 12.4762 | 0.0021 | 0.0106 |
| Novispirillum | 123.5714 | 0.0022 | 0.0106 |
| Coriobacteriaceae | 0.2296 | 0.0031 | 0.0133 |
| Clostridiales incertae sedis XIV | 0.4402 | 0.0046 | 0.0181 |
| Erysipelotrichaceae | 4.5515 | 0.0072 | 0.0260 |
| Enterobacteriaceae | 194.6429 | 0.0293 | 0.0672 |
| Desulfovibrionaceae | 4.8052 | 0.0339 | 0.0729 |
| **Genus** |  |  |  |
| Bacteroides | 10.9711 | 0.0001 | 0.0046 |
| Clostridium | 316.8571 | 0.0002 | 0.0046 |
| Turicibacter | 41.8571 | 0.0002 | 0.0046 |
| Sporacetigenium | 22.1429 | 0.0007 | 0.0092 |
| Anaerovorax | 5.3257 | 0.0011 | 0.0114 |
| Slackia | 0.1517 | 0.0017 | 0.0145 |
| Coprococcus | 0.1608 | 0.0021 | 0.0145 |
| Campylobacter | 12.4762 | 0.0021 | 0.0145 |
| Robinsoniella | 98.0952 | 0.0026 | 0.0157 |
| Lysinibacillus | 121.5714 | 0.0030 | 0.0160 |
| Olsenella | 0.1652 | 0.0040 | 0.0197 |
| Moryella | 0.0845 | 0.0045 | 0.0197 |
| Blautia | 0.4421 | 0.0046 | 0.0197 |
| Subdoligranulum | 0.2290 | 0.0046 | 0.0197 |
| Adlercreutzia | 0.2849 | 0.0054 | 0.0217 |
| Akkermansia | 14.5943 | 0.0097 | 0.0329 |
| Acetivibrio | 0.4297 | 0.0111 | 0.0349 |
| Persicirhabdus | 0.2183 | 0.0133 | 0.0403 |
| Escherichia/Shigella | 304.7143 | 0.0334 | 0.0801 |
| **OTU** |  |  |  |
| Otu000002- unclassified Verrucomicrobiaceae | 0.0727 | 0.0068 | 0.0932 |
| Otu000003- unclassified Ruminococcaceae | 0.1907 | 0.0002 | 0.0184 |
| Otu000006- Akkermansia | 17.9798 | 0.0031 | 0.0647 |
| Otu000013- Subdoligranulum | 0.0994 | 0.0004 | 0.0286 |
| Otu000020- unclassified Lachnospiraceae | 0.2856 | 0.0097 | 0.0981 |
| Otu000022- unclassified Ruminococcaceae | 0.2197 | 0.0046 | 0.0760 |
| Otu000023- unclassified Lachnospiraceae | 0.0321 | 0.0002 | 0.0184 |
| Otu000032- Olsenella | 0.1674 | 0.0020 | 0.0596 |
| Otu000033- unclassified Ruminococcaceae | 0.1552 | 0.0046 | 0.0760 |
| Otu000035- Bacteroides | 18.6180 | 0.0010 | 0.0356 |
| Otu000040- unclassified Lachnospiraceae | 0.0932 | 0.0063 | 0.0916 |
| Otu000041- unclassified Ruminococcaceae | 0.2349 | 0.0002 | 0.0184 |
| Otu000042- Campylobacter | 12.3977 | 0.0007 | 0.0286 |
| Otu000043- unclassified Lachnospiraceae | 0.1308 | 0.0097 | 0.0981 |
| Otu000044- unclassified Bacteria | 0.2066 | 0.0046 | 0.0760 |
| Otu000048- unclassified Clostridiales | 0.1172 | 0.0069 | 0.0932 |
| Otu000071- Coprococcus | 0.1974 | 0.0012 | 0.0405 |
| Otu000072- Blautia | 0.1575 | 0.0015 | 0.0478 |
| Otu000076- unclassified Clostridiales | 0.1526 | 0.0093 | 0.0981 |
| Otu000080- unclassified Lachnospiraceae | 0.1496 | 0.0095 | 0.0981 |
| Otu000086- unclassified Lachnospiraceae | 0.1972 | 0.0004 | 0.0286 |
| Otu000087- unclassified Lachnospiraceae | 29.1969 | 0.0036 | 0.0681 |
| Otu000097- unclassified Clostridiales | 0.0188 | 0.0037 | 0.0681 |
| Otu000111- unclassified Ruminococcaceae | 0.1819 | 0.0060 | 0.0916 |
| Otu000113- unclassified Lachnospiraceae | 0.1153 | 0.0010 | 0.0356 |
| Otu000115- unclassified Lachnospiraceae | 0.2179 | 0.0038 | 0.0688 |
| Otu000117- unclassified Lachnospiraceae | 0.2305 | 0.0093 | 0.0981 |
| Otu000120- unclassified Clostridiales | 0.1863 | 0.0069 | 0.0932 |
| Otu000122- unclassified Lachnospiraceae | 0.2050 | 0.0002 | 0.0184 |
| Otu000129- unclassified Lachnospiraceae | 262.0170 | 0.0002 | 0.0184 |
| Otu000170- unclassified Ruminococcaceae | 0.0618 | 0.0063 | 0.0916 |
| Otu000171- unclassified Lachnospiraceae | 0.1689 | 0.0007 | 0.0286 |
| Otu000172- Clostridium | 160.5891 | 0.0002 | 0.0184 |
| Otu000174- unclassified Clostridiales | 5.5544 | 0.0021 | 0.0596 |
| Otu000181- Alistipes | 7.0616 | 0.0071 | 0.0932 |
| Otu000182- unclassified Lachnospiraceae | 38.6121 | 0.0007 | 0.0286 |
| Otu000208- unclassified Firmicutes | 134.0252 | 0.0002 | 0.0184 |
| Otu000213- unclassified Lachnospiraceae | 0.1192 | 0.0044 | 0.0760 |
| Otu000214- unclassified Novispirillum | 128.8895 | 0.0073 | 0.0932 |
| Otu000215- Anaerovorax | 0.2497 | 0.0008 | 0.0286 |
| Otu000216- unclassified Clostridiales | 0.1762 | 0.0026 | 0.0647 |
| Otu000220- Blautia | 0.2743 | 0.0071 | 0.0932 |
| Otu000222- unclassified Clostridiales | 49.5734 | 0.0010 | 0.0356 |
| Otu000239- unclassified Clostridiales | 9.7761 | 0.0008 | 0.0286 |
| Otu000240- Ruminococcus | 0.1756 | 0.0037 | 0.0681 |
| Otu000249- unclassified Bacteria | 0.2250 | 0.0063 | 0.0916 |
| Otu000265- unclassified Ruminococcaceae | 7.0157 | 0.0026 | 0.0647 |
| Otu000267- Lysinibacillus | 122.7206 | 0.0030 | 0.0647 |
| Otu000268- unclassified Lachnospiraceae | 0.7244 | 0.0082 | 0.0981 |
| Otu000288- unclassified Alphaproteobacteria incertae sedis | 75.6603 | 0.0004 | 0.0286 |
| Otu000298- Anaerovorax | 3.3106 | 0.0031 | 0.0647 |
| Otu000303- unclassified Peptostreptococcaceae | 113.1259 | 0.0008 | 0.0286 |
| Otu000332- unclassified Ruminococcaceae | 15.0657 | 0.0074 | 0.0933 |
| Otu000335- Acetanaerobacterium | 27.6320 | 0.0002 | 0.0184 |
| Otu000350- unclassified Ruminococcaceae | 0.1758 | 0.0087 | 0.0981 |
| Otu000361- unclassified Lachnospiraceae | 0.3216 | 0.0066 | 0.0932 |
| Otu000364- unclassified Clostridiales | 5.8078 | 0.0039 | 0.0701 |
| Otu000375- Coprobacillus | 31.2396 | 0.0106 | 0.0981 |
| Otu000385- Clostridium | 74.0191 | 0.0002 | 0.0184 |
| Otu000386- unclassified Ruminococcaceae | 0.2431 | 0.0063 | 0.0916 |
| Otu000402- unclassified Ruminococcaceae | 0.2641 | 0.0005 | 0.0286 |
| Otu000443- unclassified Firmicutes | 2.0916 | 0.0073 | 0.0932 |
| Otu000448- Subdoligranulum | 19.4235 | 0.0037 | 0.0681 |
| Otu000449- unclassified Lachnospiraceae | 0.2049 | 0.0037 | 0.0681 |
| Otu000454- unclassified Alphaproteobacteria incertae sedis | 6.4219 | 0.0106 | 0.0981 |
| Otu000461- unclassified Ruminococcaceae | 0.3091 | 0.0063 | 0.0916 |
| Otu000481- Lachnospiraceae unclassified | 0.4540 | 0.0088 | 0.0981 |
| Otu000489- unclassified Clostridiales | 7.1914 | 0.0049 | 0.0785 |
| Otu000492- Blautia | 0.3692 | 0.0063 | 0.0916 |
| Otu000502- unclassified Clostridiales | 6.1207 | 0.0036 | 0.0681 |
| Otu000508- Robinsoniella | 47.9776 | 0.0030 | 0.0647 |
| Otu000516- Clostridium | 46.6829 | 0.0030 | 0.0647 |
| Otu000520- unclassified Ruminococcaceae | 6.9125 | 0.0020 | 0.0596 |
| Otu000534- Turicibacter | 39.3835 | 0.0002 | 0.0184 |
| Otu000586- Robinsoniella | 34.3185 | 0.0030 | 0.0647 |
| Otu000589- unclassified Clostridiales | 32.4840 | 0.0030 | 0.0647 |
| Otu000607- unclassified Clostridiales | 22.1976 | 0.0008 | 0.0286 |
| Otu000608- Adlercreutzia | 0.6828 | 0.0044 | 0.0760 |
| Otu000673- unclassified Ruminococcaceae | 0.4775 | 0.0087 | 0.0981 |
| Otu000705- unclassified Lachnospiraceae | 16.9863 | 0.0008 | 0.0286 |
| Otu000735- Clostridium | 20.9061 | 0.0008 | 0.0286 |
| Otu000757- Sporacetigenium | 19.3525 | 0.0008 | 0.0286 |
| Otu000768- Lachnospiraceae unclassified | 0.4357 | 0.0087 | 0.0981 |
| Otu000773- unclassified Ruminococcaceae | 3.6838 | 0.0073 | 0.0932 |
| Otu000774- Clostridium | 18.0468 | 0.0002 | 0.0184 |
| Otu000790- unclassified Bacteria | 3.8485 | 0.0106 | 0.0981 |
| Otu000800- Subdoligranulum | 14.5628 | 0.0106 | 0.0981 |
| Otu000806- unclassified Ruminococcaceae | 6.2743 | 0.0106 | 0.0981 |
| Otu000813- unclassified Bacteria | 8.8311 | 0.0106 | 0.0981 |
| Otu000848- Robinsoniella | 14.9686 | 0.0106 | 0.0981 |
| Otu000870- unclassified Lachnospiraceae | 4.8854 | 0.0026 | 0.0647 |
| Otu000885- Clostridium | 14.1464 | 0.0106 | 0.0981 |
| Otu000921- unclassified Clostridiales | 3.0119 | 0.0106 | 0.0981 |
| Otu000973- unclassified Ruminococcaceae | 0.0727 | 0.0068 | 0.0932 |
| Otu000990- Robinsoniella | 0.1907 | 0.0002 | 0.0184 |
| Otu001030- Anaerovorax | 17.9798 | 0.0031 | 0.0647 |
| Otu001132- unclassified Clostridiales | 0.0994 | 0.0004 | 0.0286 |
| Otu001312- unclassified Bacteria | 0.2856 | 0.0097 | 0.0981 |
| Otu001323- Robinsoniella | 0.2197 | 0.0046 | 0.0760 |
| Otu001369- Robinsoniella | 0.0321 | 0.0002 | 0.0184 |
| Otu001422- unclassified Clostridiales | 0.1674 | 0.0020 | 0.0596 |
| Otu001455- Dorea | 0.1552 | 0.0046 | 0.0760 |
| Otu001507- unclassified Ruminococcaceae | 18.6180 | 0.0010 | 0.0356 |
| Otu001542- unclassified Clostridiales | 0.0932 | 0.0063 | 0.0916 |
| Otu002530- Anaerovorax | 0.2349 | 0.0002 | 0.0184 |
| Otu002777- unclassified Clostridiales | 12.3977 | 0.0007 | 0.0286 |
